# Has the supply of care beds for older adults become more equal across Chinese provinces? Spatial distribution dynamics and convergence evidence from a thirteen-year panel

**DOI:** 10.3389/fpubh.2026.1956678

**Published:** 2026-10-14

**Authors:** Bei Wang, Hong Chen

**Affiliations:** 1 School of Economics and Trade, Hubei Open University, Wuhan, China; 2 School of Economics and Management, Wuhan University, Wuhan, China

**Keywords:** China, convergence, Dagum Gini decomposition, care beds for older adults, Markov chain, regional disparities, spatial autocorrelation

## Abstract

**Introduction:**

Equalizing basic older adult care services is a core objective of China’s response to population aging, yet whether the provision of older adult care bed supply has become more equal across provinces, and how its spatial structure has evolved, remains unclear. We ask how large provincial disparities in bed supply are and where they originate, whether supply is spatially clustered, how the distribution has evolved, and whether gaps converge.

**Methods:**

Using a balanced panel of 31 provinces over 2011–2023 (403 observations), bed supply is measured as beds in registered institutions and community facilities per 1,000 persons aged 60 and over. The analysis combines the Dagum Gini decomposition, global and local Moran’s I under three weight matrices, kernel densities, ordinary and spatial Markov chains, σ- and β-convergence tests with spatial-lag checks, the Phillips–Sul log t test, and system GMM.

**Results:**

The main findings are as follows: (i) the Gini coefficient fell from 0.256 in 2012 to 0.125 in 2023 (−51.1%), driven by the collapse of the net between-region component from 42.6 to 13.9% of total inequality, so that by 2023 transvariation (51.9%) rather than between-region gaps dominated; (ii) bed supply was significantly spatially clustered in 2011–2014 (Moran’s I up to 0.478, *p* < 0.001; a Yangtze River Delta high–high cluster), but clustering dissolved from 2015 onward; (iii) the distribution stayed unimodal and Markov mobility was bidirectional under relative and absolute classifications; (iv) the east fell from the highest to the lowest regional mean by 2023, led by sharp declines in Zhejiang, Hainan, Guangdong and Beijing; (v) β-convergence held in every specification and was unaffected by a spatial-lag term; the log t test did not reject national convergence (*t* = 0.958) but rejected it within the east (*t* = −4.445); system GMM implies about 23% per year, an upper bound.

**Discussion:**

Equalization of bed supply has advanced substantially and dissolved earlier spatial clustering, but coincides with declining relative provision as the older population outpaces bed expansion. The eastern decline may reflect a shift toward service-based care—eastern community service volume doubled over 2020–2023—but this expansion was not concentrated where beds fell, and mechanisms remain unidentified.

## 1 Introduction

China is aging at a pace with few historical precedents. The population aged 60 and over reached 297 million by 2023, and providing this population with accessible, affordable care has become a first-order public health and fiscal challenge. The State Council’s Plan for the Development of the National Undertakings on Aging and the Elderly Care Service System during the 14th Five-Year Plan Period (Guofa [2021] No. 35) responds by mandating an integrated system that coordinates home-, community- and institution-based care, and by elevating the equalization of basic older adult care services to a core policy objective (1). The strategy builds on the National Medium- and Long-Term Plan for Actively Responding to Population Aging (2) and on a decade of institution building whose policy landscape has been reviewed in The Lancet (3). By the end of 2023 China reported 404,000 older adult care institutions and facilities with 8.23 million beds, of which 5.17 million were in registered institutions and 3.06 million in community-based facilities (4).

Aggregate expansion of this magnitude invites an optimistic reading. Yet the policy-relevant quantity is not the stock of beds but supply relative to the population served. Earlier assessments placed China at roughly 24 beds per 1,000 older persons in 2014, well short of the 35–40 bed target embedded in national planning (5). Because the older population itself has grown rapidly, absolute expansion and relative improvement can move in opposite directions. Moreover, the 297 million older persons are far from evenly distributed: in 2023 the share of population aged 65 and over ranged from 6.1% in Tibet to 21.1% in Liaoning, so that the same 30 beds per 1,000 older persons corresponds to very different absolute service populations, and provincial differences in aging can amplify or attenuate the substantive meaning of bed-supply disparities. This is why we normalize by the older population rather than by total population, and why the spatial pattern of relative supply, not merely its average, is the object of study.

Regional inequality has been a central concern of Chinese economic geography for three decades. Geographers have documented the rise, persistence and partial reversal of coastal–interior gaps in income and development (6–8) and have traced their sources to policy regimes as much as to endowments (9). A parallel literature on spatial equity examines whether public facilities are distributed fairly relative to need, emphasizing accessibility as the mechanism through which location translates into use (10–12). Applied to older adult care, this literature has mapped the accessibility and equity of residential care facilities within Chinese cities (13, 14) and, most recently, the spatial pattern of facility occupancy and community meal-assistance services in Beijing (15, 16). The present study positions provincial bed supply within this geographical frame: it treats the spatial allocation of older adult care facilities as an equalization problem, asks whether provincial differences are spatially structured or spatially random, and tests whether the equalization process has altered that structure.

Three further strands of literature speak to these questions, each leaving a gap this paper addresses. The first documents inequality in care resource allocation. Yang and co-authors, using the China Civil Affairs Statistical Yearbook and benchmarking resources against the older population, find substantial urban–rural inequality and show that the eastern region combines the highest resource levels with the greatest internal dispersion (17). This line of work establishes the measurement agenda but is essentially static. The second strand examines efficiency and spatial patterns, evaluating the joint equity and efficiency of care resource allocation over 2009–2020 (18), mapping the spatial inequity of care institutions with point-of-interest data (19), and assessing the equity of older-adult resource allocation in a 2020 cross-section (20). These studies enrich the spatial picture but stop short of formal convergence analysis, and their samples end before the recent period in which, as we show, the regional hierarchy reversed. The third strand applies distribution-dynamics methods to regional outcomes in China, including the health of older adults (21) and broader development indicators (22, 23); however, these tools have not yet been brought to bear on older adult care bed supply, none of the applications confronts the Nickell bias that inflates convergence speeds in dynamic panels with fixed effects (24), and none integrates the spatial econometric tools—spatial convergence models, spatial Markov chains and local indicators of spatial association—that geographers have developed precisely for such questions (25–27).

Two clarifications about scope and regional units are needed at the outset. First, the study examines older adult care bed supply—beds in registered institutions and community-based facilities as published by the Ministry of Civil Affairs—and not the broader older adult care service system, which also comprises home-based services and long-term care insurance coverage that beds do not measure. Second, we adopt the eastern–central–western three-belt classification as the primary regional grouping because it is the unit at which national equalization policy, fiscal transfers and planning targets are formulated and evaluated; it is a product of macro-regional strategy rather than a geographically homogeneous partition, provinces within each belt differ in terrain, density and fiscal capacity, and the Northeast is treated separately in a four-region robustness check. Because policy-defined belts may not coincide with the spatial structure of the data, we complement the belt analysis with contiguity-based spatial statistics that let neighborhood effects and cross-border similarity emerge from the data themselves.

The marginal contributions of this study are as follows: (i) it constructs, to our knowledge, the longest available provincial panel of officially published bed supply per 1,000 older persons (2011–2023), verifies its numerator and denominator against national totals, and documents the nature and spatial incidence of the 2018 statistical revision; (ii) it integrates the Dagum Gini decomposition, kernel density estimation, Markov chains and σ-/β-convergence testing with global and local spatial autocorrelation analysis, spatial Markov chains and spatial-lag convergence models, and disciplines the convergence estimates with two-step system GMM and the Phillips–Sul log t test; (iii) it documents that bed supply was significantly spatially clustered in the early 2010s—with a Yangtze River Delta high–high cluster and a southern low–low cluster—and that this clustering dissolved as equalization proceeded, a finding that reframes convergence as a spatial de-clustering process; and (iv) it documents a structural reversal of the regional hierarchy, identifies which eastern provinces drove it, and establishes that equalization of bed supply has coincided with a plateau and recent decline in relative provision.

The remainder of the paper proceeds as follows. Section 2 develops the conceptual framework and states four testable propositions. Section 3 describes the data, study area and methods. Section 4 presents stylized facts. Section 5 reports the empirical results, including robustness checks. Section 6 discusses mechanisms, policy implications and limitations, and Section 7 concludes.

## 2 Conceptual framework and propositions

Older adult care beds are a quasi-public good provided locally. The theory of fiscal federalism implies that decentralized provision tracks local fiscal capacity and preferences (28), while residential sorting links provision to the composition of local demand (29). Within this setting, three groups of forces shape provincial bed supply. On the demand side, population aging scales the need for care; the economics of long-term care establishes substitution between institutional and informal family care, so that urbanization, migration and shrinking households shift demand from families toward formal facilities (30, 31); and rising incomes convert latent need into effective demand while attracting private capital into supply. On the supply side, bed construction and operation depend heavily on government funding, making local fiscal capacity the binding constraint; medical resources complement care provision under China’s integrated medical-and-older adult care model; and because care is a labor-intensive stagnant sector, its relative cost rises systematically with development—the Baumol effect—so that land and labor costs restrain expansion precisely where development is most advanced (32). On the institutional side, the national equalization mandate, redistributive fiscal transfers and quantified planning targets anchor the behavior of lagging provinces (33).

Geography enters this framework in two ways. First, spatial equity and accessibility theory hold that the welfare value of a facility depends on where it is relative to those who need it (10–12); at the provincial scale this implies that supply should be assessed relative to the local older population and that adjacent provinces may share unobserved conditions—terrain, transport networks, labor markets, policy diffusion—that generate spatial dependence. Second, spatial convergence theory recognizes that growth in one unit may spill over to its neighbors through fiscal demonstration effects, cross-border demand and inter-provincial migration, so that convergence can be spatially structured rather than independent across units (25). Whether such dependence is present is an empirical question that the spatial tests below address directly.

Convergence of provincial gaps can then arise through two channels. The neoclassical channel operates through diminishing returns: where beds are scarce, additional beds are cheapest to add and most valuable, so low-baseline provinces grow faster (34). The institutional channel operates through policy: transfers and targets compress the distribution from above and below. The Baumol cost mechanism adds a third force—cost-driven deceleration at the top—implying that observed convergence may reflect slowdown among leaders as much as catch-up among laggards. A fourth possibility, specific to the bed indicator, is that developed provinces substitute toward home- and community-based models that beds do not capture; this would produce an apparent decline in measured supply without a decline in care capacity.

The framework yields four testable propositions. P1: provincial disparities in bed supply per older person decline over the study period, and the decline originates mainly in the erosion of between-region differences. P2: bed supply exhibits positive spatial autocorrelation in the early period, and equalization weakens that autocorrelation over time. P3: the cross-provincial distribution remains unimodal—institutional anchoring prevents club formation—while mobility is bidirectional, reflecting both catch-up and deceleration. P4: β-convergence holds in absolute and conditional form, is robust to the inclusion of spatial-lag terms, and the bias-corrected convergence rate from system GMM is materially lower than fixed-effects estimates.

## 3 Materials and methods

### 3.1 Study area, scale and data

The study area comprises all 31 provincial-level administrative units of mainland China (22 provinces, five autonomous regions and four centrally administered municipalities). The provincial scale is chosen for three reasons. It is the level at which the bed-supply indicator is officially published on a consistent basis for the full period; prefectural data for this indicator are not published on a consistent national basis for all years. It is the level at which equalization policy is formulated and fiscal transfers are allocated, so that provincial disparities are the disparities the policy seeks to reduce. And it is the level at which the three-belt classification is defined. The 31 units are highly heterogeneous: they span coastal and inland locations, plains, plateaus and mountain regions, population densities from about 3 to over 3,800 persons per square kilometer, urbanization rates from 22 to 94%, and fiscal self-sufficiency ratios from 7 to 93% (Table 1). This heterogeneity is precisely what the spatial and regional analyses exploit; its cost is that within-province variation is not observed, a limitation discussed in Section 6.

**Table 1:** Descriptive statistics.

Variable	*N*	Mean	SD	Min	Max
Beds per 1,000 older persons	403	26.87	9.54	3.92	61.90
Aging rate (65+, %; description only)	403	11.29	3.20	4.82	21.06
Urbanization rate (%)	372	58.79	13.21	22.22	94.15
Per capita GRP (log)	403	10.93	0.46	9.71	12.21
Urban disposable income (log)	403	10.41	0.35	9.62	11.40
Per capita fiscal expenditure (log)	403	9.51	0.49	8.41	11.25
Fiscal self-sufficiency (%)	403	47.58	19.66	6.93	93.14
Child dependency ratio (%)	403	23.39	6.41	9.88	38.38

Urbanization is unavailable for 2020 (census year) in all 31 provinces.

The outcome is the officially published indicator of older adult care beds per 1,000 older persons from the China Civil Affairs Statistical Yearbook, accessed through the EPS data platform. The numerator is beds in all older adult care institutions and facilities—registered institutions plus community-based facilities providing residential or day care—as defined by the Ministry of Civil Affairs; in 2023 these comprised 5.17 million and 3.06 million beds, respectively, (4). The indicator therefore measures bed-based supply and excludes home-based services and long-term care insurance coverage. The denominator is the population aged 60 and over rather than 65 and over, verified against national totals: 6.727 million beds in 2015 divided by 222 million persons aged 60 and over yields 30.3 beds per 1,000, matching the panel mean of 30.32, whereas a 65-and-over denominator would imply 46.7. The indicator is taken as published; the older population it uses is that of the civil affairs statistical system, which we cannot decompose into resident and registered components, and we note this in the limitations. Two sub-series covering 2011–2016 and 2017–2023 were concatenated; cross-validation on overlapping years showed exact agreement. The panel is balanced with 403 province-year observations and no missing values on the outcome.

A revision to the civil affairs statistical system took effect in 2018. The Ministry’s 2018 statistical communiqué states that, from 2018, older adult care institutions and their beds no longer include honored-veteran rehabilitation hospitals, demobilized-soldier sanatoria and military retiree centers (35); the revision was thus a change in statistical coverage rather than in the definition of a bed or in population estimation. The national mean fell from 29.56 in 2017 to 27.20 in 2018 and the Gini coefficient rose from 0.156 to 0.183. Section 5.8 shows that the incidence of the revision was spatially uneven: the 2017–2018 change ranged from −52.6% in Tibet to +8.7% in Anhui, was significantly larger in the west (mean −14.1%) than in the center (−1.0%), and was itself spatially clustered. Because the revision removed a category of facilities rather than redefining the remaining ones, the 2011–2017 and 2018–2023 sub-series are each internally consistent, and we treat the break with four measures: a revision dummy in panel specifications, separate estimation of every distributional and convergence statistic for the two sub-periods, explicit annotation of the break in figures, and a check that kernel density and Markov results are unchanged within each sub-period.

Covariates are drawn from the China Statistical Yearbook and the Finance Yearbook of China: per capita general public budget expenditure (log), urban resident disposable income (log), the urbanization rate, per capita gross regional product (log), and fiscal self-sufficiency (the ratio of local budget revenue to expenditure). Monetary variables are deflated. Following the reviewers’ advice, the share of population aged 65 and over is not used as an explanatory variable, because the outcome is normalized by the older population and any such variable would be mechanically related to it; it is reported in Table 1 for description only. As a demand-side indicator that does not overlap with the denominator, the child dependency ratio (population aged 0–14 relative to population aged 15–64, from the annual population sampling survey; linearly interpolated for the 2020 census year) is used instead. The urbanization rate is unavailable for 2020 in every province, because the annual population sampling survey was replaced by the national census in that year; the 31 missing values are therefore concentrated in a single census year and are not associated with region. Specifications that include urbanization use 372 observations, and all such specifications are re-estimated without it on the full 403 observations. Provinces are grouped into eastern, central and western regions under the conventional three-belt classification; a four-region classification separating the Northeast is used in robustness.

### 3.2 Disparity measurement and distribution dynamics

The Dagum Gini coefficient decomposes total inequality into within-region inequality, net between-region inequality and transvariation intensity (36):


$$G=\frac{\Sigma_{j=1}^{k}\Sigma_{h=1}^{k}\;\Sigma_{i=1}^{\mathrm{nj}}\Sigma_{r=1}^{\mathrm{nh}}|y_{\mathrm{ji}}-y_{\mathrm{hr}}|}{2n^{2}\mu }$$

Gaussian kernel densities characterize the location, shape and dispersion of the distribution over time. Markov transition matrices classify provinces into four states relative to the national mean (cut-offs 0.75, 1.00, 1.25) and estimate one-year transition probabilities. Because the national mean is not stationary over the period, two alternative classification schemes are used as robustness checks: fixed thresholds set at the quartiles of the 2011 distribution (12.8, 17.2 and 23.3 beds), and absolute thresholds of 20, 30 and 40 beds per 1,000 older persons. The ergodic distribution and the second-largest eigenvalue of the transition matrix summarize the long-run distribution and the speed of approach to it.

### 3.3 Spatial autocorrelation, local clusters and spatial Markov chains

Spatial dependence is assessed with three spatial weight matrices, all row-standardized: (i) a Queen contiguity matrix based on shared provincial boundaries, in which Hainan is linked to Guangdong by convention (average 4.45 neighbors per province); (ii) an inverse-distance matrix with elements 1/d_ij_, where d_ij is the great-circle distance between provincial capitals; and (iii) an inverse-squared-distance matrix with elements 1/d_ij_^2^. Global Moran’s I is computed for every year from 2011 to 2023 under each matrix, with inference based on 9,999 random permutations. Local indicators of spatial association (LISA) (27) identify high–high, low–low, high–low and low–high clusters under the contiguity matrix at the 5% level for 2011, 2013, 2017 and 2023; no correction for multiple comparisons across the 31 provinces is applied, and the LISA results are used descriptively to locate clusters rather than as formal tests. A spatial Markov chain (26) conditions the transition matrix on the initial spatial lag of each province, using the same four states for provinces and for their neighbors, and tests the homogeneity of the conditional matrices with the Q and likelihood-ratio statistics. Finally, the spatial lag of the lagged level, W·ln y_i,t − 1_, is added to the panel convergence model as a robustness check on spatial spillovers.

### 3.4 Convergence tests and dynamic panel estimation

Following the classical approach to convergence analysis (37), σ-convergence is tracked with the coefficient of variation and the Theil index, and absolute β-convergence regresses average annual growth on the log initial level:


$$\frac{1}{T}\ln(\frac{\mathrm{yi},t+T}{\mathrm{yi},t})=\alpha +\beta \ln(\mathrm{yi},t)+\mathrm{\varepsilon i}$$

where T is the length of the interval in years: *T* = 12 for the full sample (2011 to 2023), *T* = 6 for 2011–2017 and *T* = 5 for 2018–2023. The implied convergence speed is s = −ln(1 + βT)/T and the half-life is ln(2)/s. Conditional convergence is estimated in panel form with covariates and fixed effects. Because dynamic panels with unit fixed effects suffer Nickell bias of order 1/T (24), the model is re-estimated by two-step system GMM (38, 39) with collapsed instruments (19 instruments for 31 groups), time dummies, and Windmeijer-corrected standard errors (40); the Arellano–Bond tests for first- and second-order serial correlation in the differenced residuals, AR(1) and AR(2), and the Hansen test of overidentifying restrictions are reported. The GMM estimate is treated as an upper-bound reference value rather than as the preferred speed, for reasons discussed in Section 5.6. The Phillips–Sul log t regression tests relative convergence without imposing a common steady state (41, 42); full regression output is provided in Appendix Table A1. A two-way fixed-effects specification with province-clustered standard errors examines correlates of bed-supply levels; these estimates are descriptive and no causal interpretation is attached. All computations were performed in Python 3.12 (pyfixest, pydynpd, esda, giddy); code is available on request.

## 4 Stylized facts

Three facts organize the empirical analysis. First, national mean bed supply nearly doubled between 2011 and 2016—from 17.85 to 30.78 beds per 1,000 older persons—then entered a plateau, easing to 29.84 by 2021 and declining to 27.27 in 2023. At no point did the mean reach the lower bound of the 35–40 bed planning target. The post-2016 decline is not a contraction of supply: absolute bed counts continued to rise; rather, the population aged 60 and over grew faster than beds were added.

Second, the regional hierarchy passed through three phases (Figure 1). In 2011 the central region led at 20.80 beds, with the east at 19.31 and the west at 14.53. Between 2013 and 2017 the east expanded aggressively and established a clear lead, peaking at 32.14 beds in 2017. After 2018 eastern supply declined continuously, reaching 26.16 beds in 2023—18.6% below its 2017 peak and, more conservatively, 10.4% below its post-revision 2018 level of 29.20—while the central and western regions consolidated at 28.38 and 27.55 beds, respectively. The maps in Figures 2A–C show the same transition province by province: in 2011 supply was low almost everywhere, with a modest coastal advantage; by 2017 a band of high supply ran from Inner Mongolia through Beijing–Tianjin to the Yangtze River Delta; by 2023 that band had dissolved into a far more uniform surface, with the highest values in Fujian, Inner Mongolia and Hubei and the lowest in Hainan, Yunnan and Liaoning—so that neither tail any longer maps onto the east–west development gradient.

**Figure 1:**
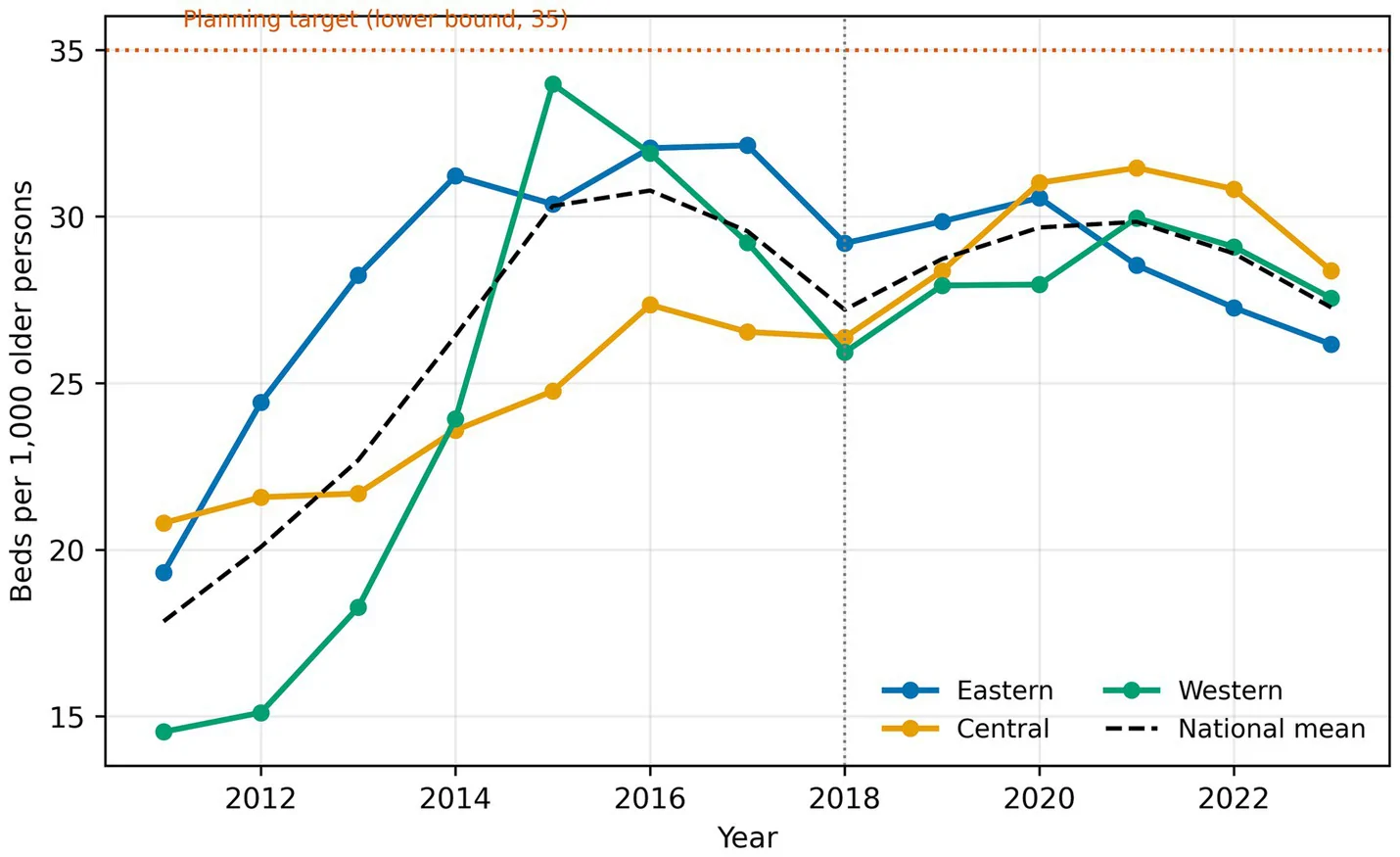
Evolution of older adult care bed supply by region, 2011–2023.

**Figure 2:**
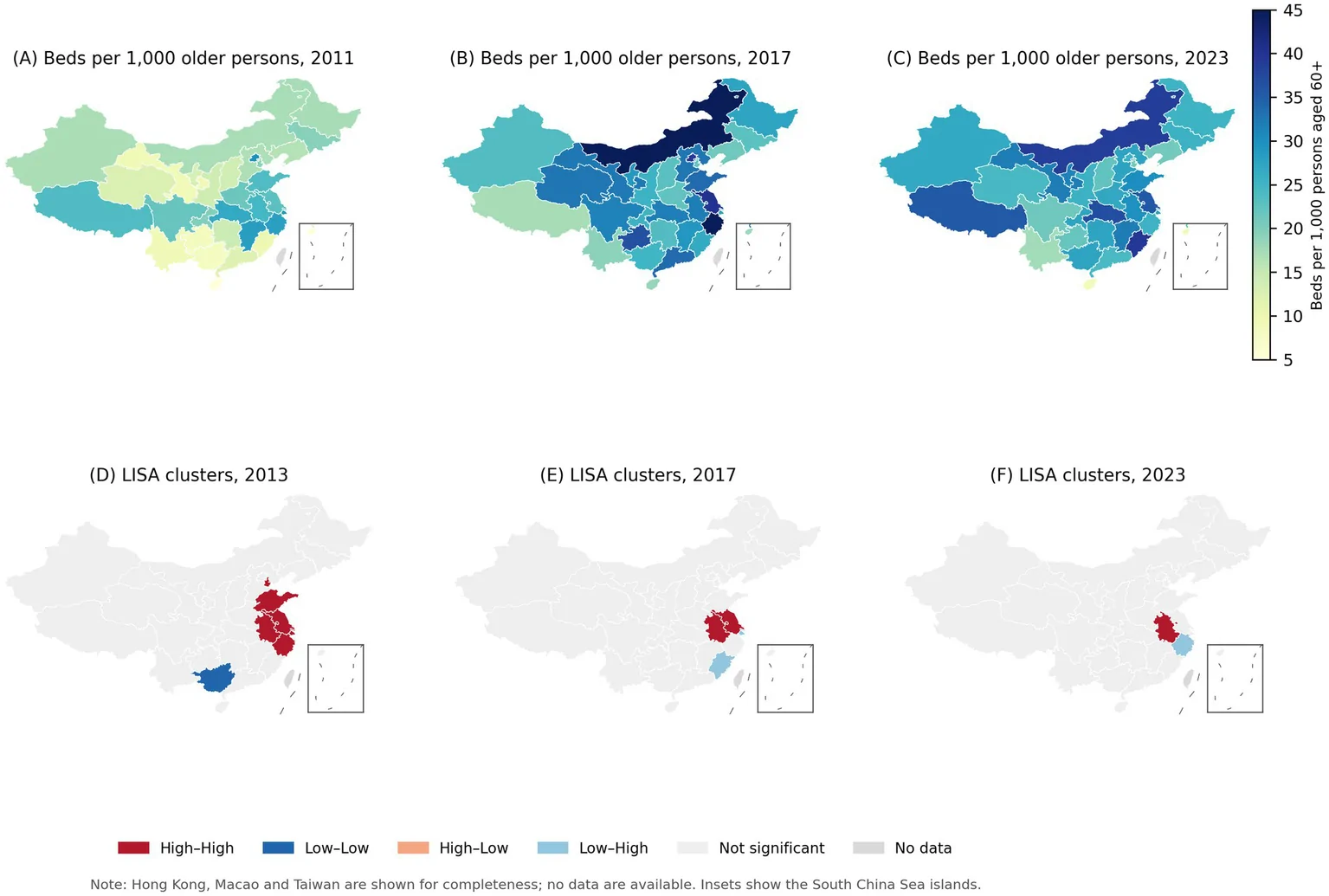
Spatial distribution of older adult care bed supply **(A–C)** and LISA cluster maps under Queen contiguity **(D–F)**. Hong Kong, Macao and Taiwan shown for completeness (no data). Base map: Natural Earth (https://www.naturalearthdata.com) (data in the public domain); national boundary in the South China Sea and Tibet drawn following the Standard Map of China, Ministry of Natural Resources (http://bzdt.ch.mnr.gov.cn; review no. GS(2019)1830).

Third, dispersion narrowed dramatically. The gap between the best- and worst-provided provinces fell from a ratio of roughly 7.7 in 2011 (61.9 versus 3.9 beds) to 4.6 in 2023 (39.6 versus 8.7). The remainder of the paper quantifies this compression, establishes its spatial structure, and tests whether it constitutes formal convergence.

## 5 Results

### 5.1 Regional disparities and their sources

Table 2 reports the disparity measures and the full Dagum decomposition. The overall Gini coefficient peaked at 0.256 in 2012 and fell to 0.125 by 2023—a cumulative decline of 51.1%. In economic terms, the expected supply gap between two randomly drawn provinces fell from 51 to 25% of the national mean. The coefficient of variation declined from 0.459 to 0.235 and the Theil index from 0.101 to 0.029; setting aside the technical increase in 2018, the decline was monotonic (Figure 3).

**Table 2:** Dagum decomposition of regional disparities, 2011–2023.

Year	Gini	Gw	Gnb	Gt	Gw (%)	Gnb (%)	Gt (%)	CV	Theil
2011	0.2222	0.0681	0.0795	0.0746	30.6	35.8	33.6	0.394	0.0796
2012	0.2557	0.0751	0.1088	0.0718	29.4	42.6	28.1	0.459	0.1013
2013	0.2279	0.0654	0.1019	0.0606	28.7	44.7	26.6	0.408	0.0803
2014	0.2036	0.0642	0.0656	0.0738	31.5	32.2	36.2	0.384	0.0668
2015	0.1938	0.0630	0.0636	0.0672	32.5	32.8	34.7	0.370	0.0613
2016	0.1677	0.0570	0.0295	0.0813	34.0	17.6	48.4	0.319	0.0464
2017	0.1555	0.0520	0.0400	0.0635	33.5	25.7	40.8	0.302	0.0406
2018	0.1830	0.0624	0.0277	0.0929	34.1	15.1	50.7	0.361	0.0607
2019	0.1562	0.0532	0.0154	0.0875	34.1	9.9	56.0	0.311	0.0447
2020	0.1462	0.0479	0.0237	0.0745	32.8	16.2	51.0	0.278	0.0379
2021	0.1269	0.0428	0.0206	0.0636	33.7	16.2	50.1	0.239	0.0304
2022	0.1269	0.0427	0.0260	0.0582	33.7	20.5	45.9	0.239	0.0302
2023	0.1252	0.0428	0.0174	0.0650	34.2	13.9	51.9	0.235	0.0287

Gw, within-region contribution; Gnb, net between-region contribution; Gt, intensity of transvariation. The 2018 increase reflects the statistical revision. CV, coefficient of variation.

**Figure 3:**
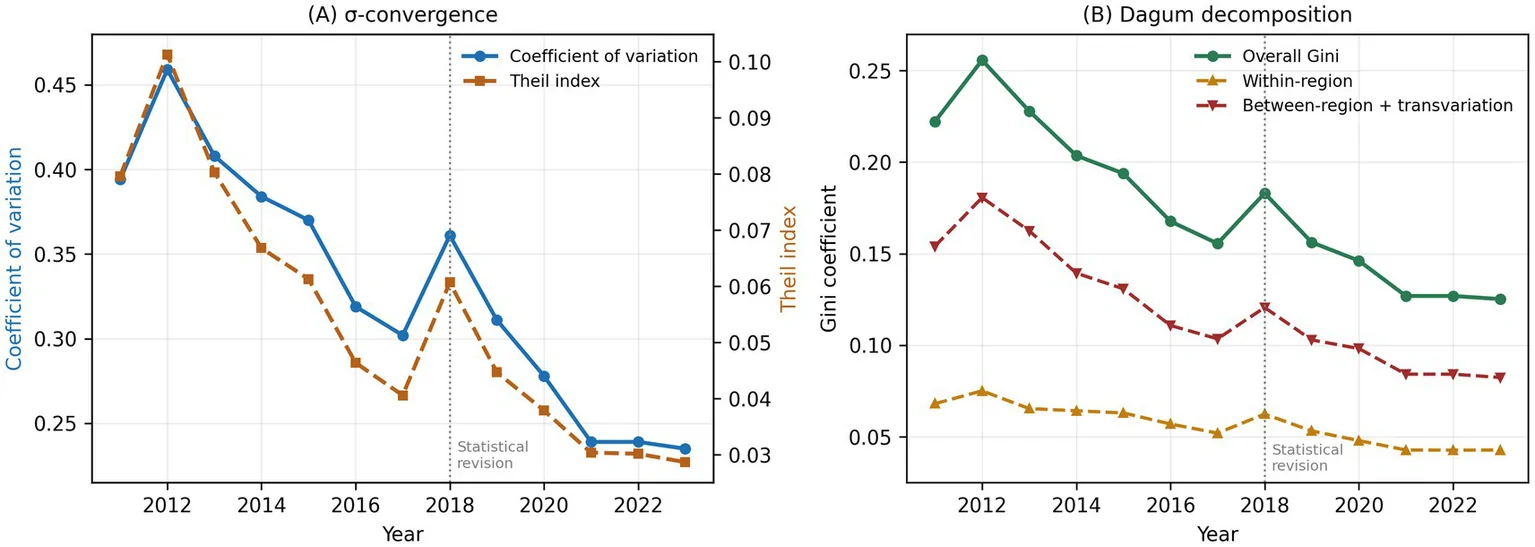
**(A,B)** σ-convergence and Dagum decomposition.

The three-way decomposition separates two distinct facts that must not be conflated. The first concerns the source of the decline: the within-region component was remarkably stable, contributing between 29 and 34% of total inequality throughout, whereas the net between-region component collapsed from 42.6% of total inequality in 2012 to 13.9% in 2023. The reduction in overall inequality was therefore driven by the erosion of between-region gaps. The second concerns the structure of the inequality that remains: by 2023 transvariation intensity—the extent to which the distributions of different regions overlap—had risen from 28.1 to 51.9% and was the dominant component, while between-region gaps had become the smallest. In other words, differences in regional means largely disappeared, and the inequality that persists in 2023 is overlap rather than hierarchy. This provides an independent statistical signature of the hierarchy reversal documented in Section 4, obtained from a decomposition that makes no reference to which region leads. Proposition P1 is supported: disparities fell, and they fell principally through the erosion of between-region gaps.

### 5.2 Spatial dependence: global and local autocorrelation

Table 3 reports global Moran’s I for every year under the three weight matrices, and Figure 4 plots the trajectory. The pattern is unambiguous and consistent across matrices. From 2011 to 2014 bed supply was significantly and positively spatially autocorrelated: under Queen contiguity Moran’s I rose from 0.339 (*p* = 0.003) in 2011 to 0.478 (*p* < 0.001) in 2013 before falling to 0.182 (*p* = 0.044) in 2014, and the inverse-distance matrices show the same rise and fall at lower magnitudes, as expected for weights that spread influence across the whole country. From 2015 onward global autocorrelation is statistically indistinguishable from zero in every year except 2020, when a modest positive value (I = 0.190, *p* = 0.032) appears under all three matrices and then recedes. By 2023 Moran’s I under contiguity is 0.048 (*p* = 0.232).

**Table 3:** Global Moran’s I of bed supply per 1,000 older persons, 2011–2023.

Year	Queen: I	*p*	Inverse distance: I	*p*	Inverse distance^2^: I	*p*
2011	0.339	0.003	0.068	0.004	0.177	0.005
2012	0.405	0.001	0.089	0.001	0.225	0.002
2013	0.478	<0.001	0.115	0.001	0.289	<0.001
2014	0.182	0.044	0.055	0.008	0.177	0.007
2015	−0.085	0.352	−0.027	0.365	−0.001	0.280
2016	−0.020	0.425	−0.031	0.411	−0.026	0.424
2017	−0.089	0.332	−0.034	0.451	−0.034	0.480
2018	0.032	0.263	−0.002	0.128	0.034	0.146
2019	−0.027	0.456	−0.016	0.235	0.000	0.284
2020	0.190	0.032	0.025	0.037	0.087	0.045
2021	0.059	0.212	0.004	0.100	0.066	0.080
2022	0.057	0.212	0.003	0.104	0.064	0.082
2023	0.048	0.232	0.000	0.128	0.058	0.101

All matrices row-standardized; *p*-values from 9,999 random permutations. Distances are great-circle distances between provincial capitals.

**Figure 4:**
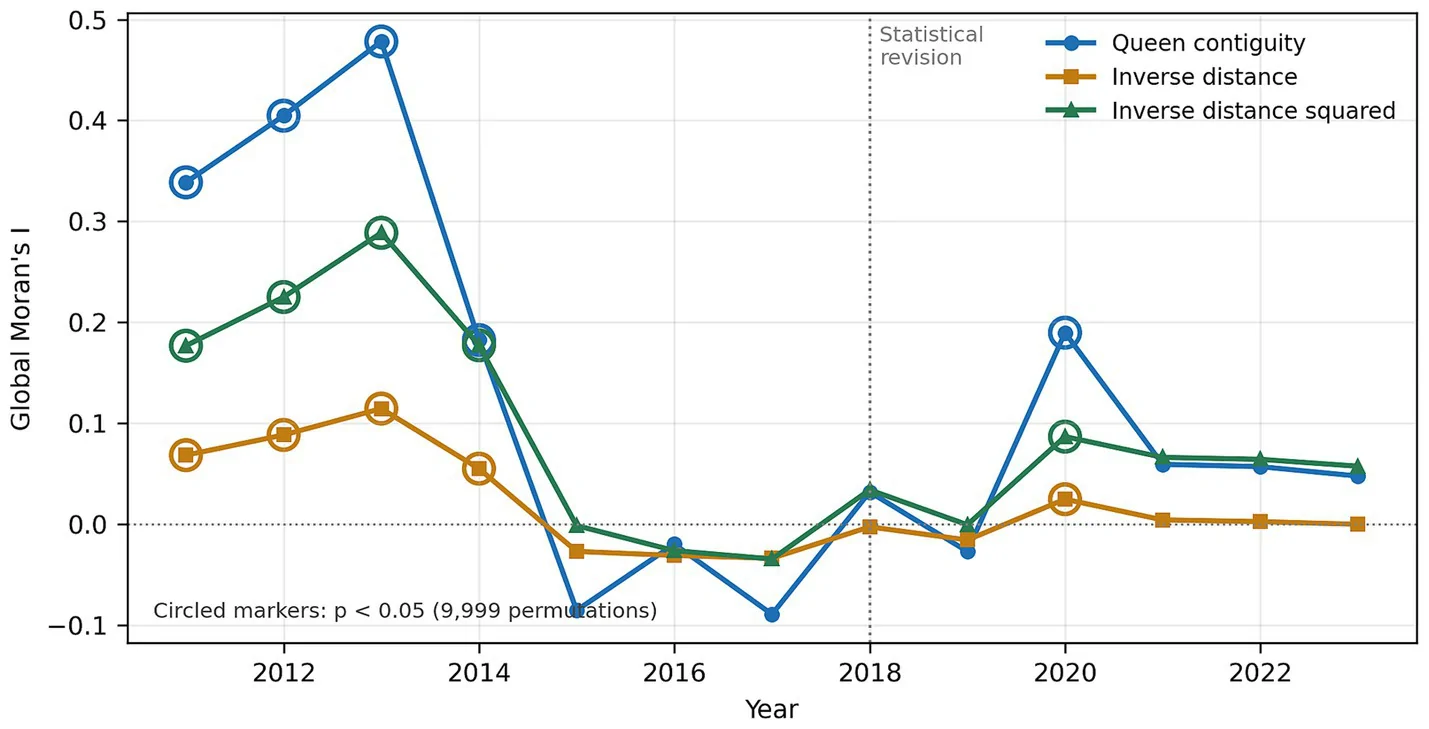
Global Moran’s I of bed supply under three spatial weight matrices, 2011–2023. Circled markers are significant at the 5% level.

Local indicators of spatial association identify where the early clustering was located and how it dissolved (Table 4; Figures 2D–F). In 2011 and 2013 a high–high cluster occupied the Yangtze River Delta and its hinterland—Shanghai, Jiangsu, Zhejiang and Anhui, joined in 2013 by Shandong and Tianjin—while a low–low cluster covered Guangdong and Guangxi in the south in 2011 (Guangxi alone in 2013). By 2017 the high–high cluster had contracted to Jiangsu and Anhui, and two coastal provinces, Shanghai and Fujian, had become low–high outliers: provinces whose own supply fell below that of their high-supply neighbors. By 2023 only Anhui remained a significant high–high location, and Zhejiang had become a low–high outlier. The progression from a compact coastal high–high cluster to isolated low–high outliers is the local counterpart of the eastern decline documented in Section 4, and it locates that decline precisely: it is the Delta’s coastal core, not its interior, that fell behind its neighbors.

**Table 4:** Significant LISA clusters (Queen contiguity, *p* < 0.05).

Year	High–High	Low–low	Low–high	Total significant
2011	Shanghai, Jiangsu, Zhejiang, Anhui	Guangdong, Guangxi	—	6
2013	Shanghai, Tianjin, Jiangsu, Zhejiang, Anhui, Shandong	Guangxi	—	7
2017	Jiangsu, Anhui	—	Shanghai, Fujian	4
2023	Anhui	—	Zhejiang	2

Local Moran’s I with 9,999 conditional permutations. No high–low clusters were significant in any year.

Taken together, the global and local results establish Proposition P2 and add a spatial dimension to the convergence story. In the early 2010s bed supply was spatially structured, with a well-defined coastal cluster of high supply and a southern cluster of low supply. Equalization did not merely narrow the gap between provinces; it dissolved the spatial clustering of that gap, so that by the end of the period the location of a province no longer predicted its supply. The transient return of autocorrelation in 2020 coincides with the census year and the first pandemic year, does not persist, and we do not attach an interpretation to it. The insignificance of global Moran’s I in recent years is therefore not a null result to be dismissed: it is the end-state of a documented de-clustering process, and it is why spatial econometric models do not alter the convergence estimates reported below.

### 5.3 Distribution dynamics and mobility

Kernel densities (Figure 5) shift rightward through 2016, consistent with the expansion phase; thereafter the mode stabilizes while the peak rises, the distribution narrows, and the right tail retracts. The distribution is unimodal in every year—no twin peaks in the sense of Quah (41) and no sign of club formation. Estimated within each sub-period, the density is unimodal at both ends of 2011–2017 (skewness rising from 0.05 to 1.35 as the eastern surge stretched the right tail) and of 2018–2023 (skewness falling from 1.02 to −0.33 as the tail retracted), so the shape conclusions do not depend on pooling across the 2018 revision.

**Figure 5:**
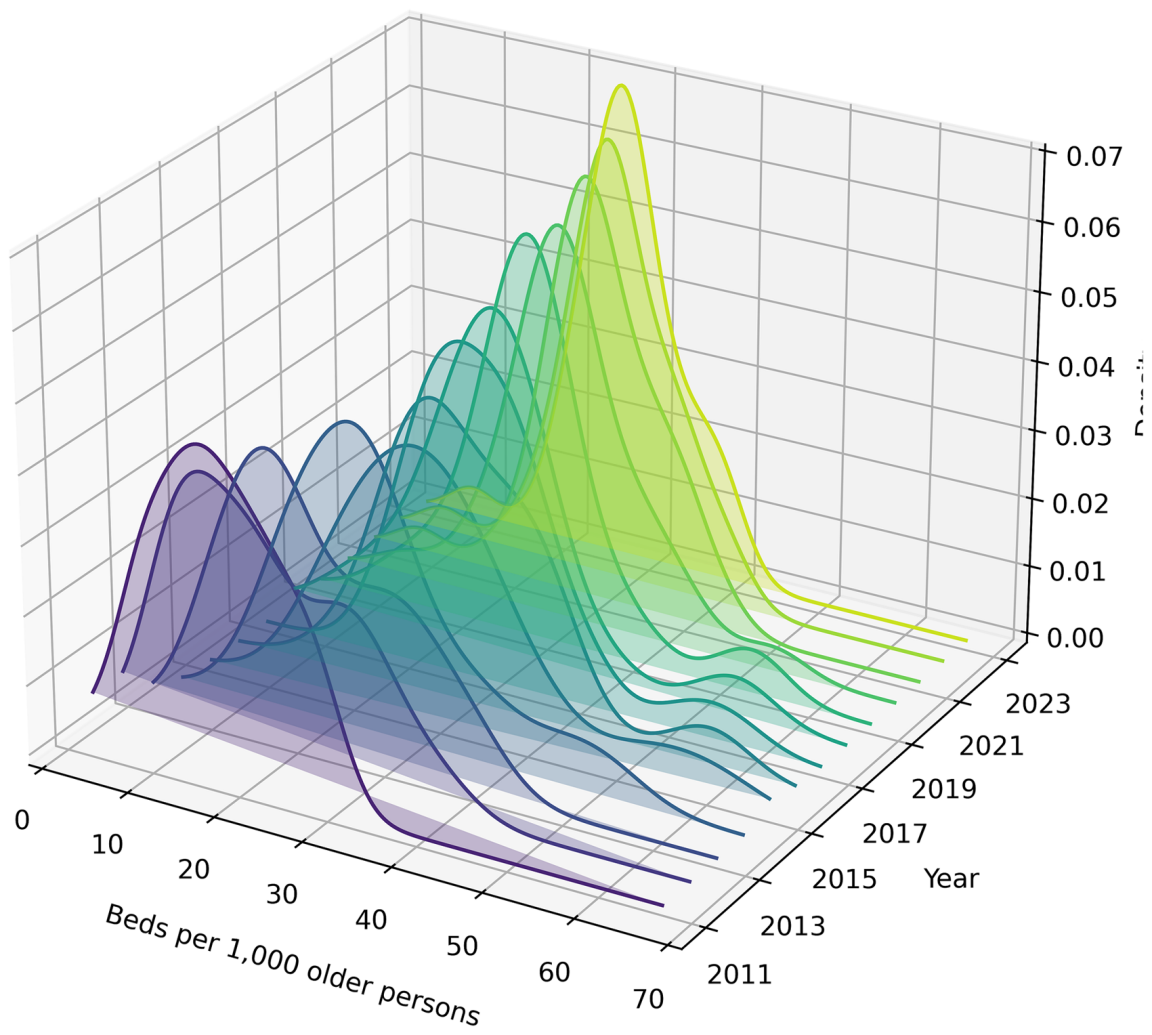
Kernel density evolution of older adult care bed supply, 2011–2023.

The Markov matrix (Table 5) shows a mean diagonal probability of 0.724: states are persistent but far from absorbing, and mobility is bidirectional—provinces in the lowest state move up with total probability 0.260 per year while provinces in the highest state move down with probability 0.247. Because the national mean rose and then fell over the period, a province could in principle change state without any change in its own supply. Table 6 therefore re-estimates the chain under two schemes that do not depend on the moving mean. Under absolute thresholds of 20, 30 and 40 beds the bidirectional pattern is, if anything, stronger (upward from the lowest state 0.284; downward from the highest 0.379). Under fixed thresholds set at the 2011 quartiles, upward moves dominate (0.393 versus 0.056), which is the expected signature of the aggregate expansion: relative to the 2011 distribution nearly every province rose in absolute terms. The two schemes thus answer different questions—the relative and absolute schemes describe position within the contemporaneous distribution, the 2011-baseline scheme describes absolute progress—and the conclusion that catch-up from below and deceleration from above occur at comparable rates in relative terms is robust. The same holds within each sub-period (Table 6, last two rows).

**Table 5:** One-year Markov transition probability matrix, 2011–2023.

Initial state	Low	Medium-low	Medium-high	High
Low	0.740	0.219	0.041	0.000
Medium-low	0.081	0.735	0.162	0.022
Medium-high	0.000	0.211	0.667	0.122
High	0.014	0.096	0.137	0.753

States defined relative to the national mean with cut-offs 0.75, 1.00, 1.25. Ergodic distribution: 0.135, 0.403, 0.285, 0.177; second-largest eigenvalue 0.773 (half-life of approach 2.7 years).

**Table 6:** Markov mobility under alternative classification schemes and sub-periods.

Scheme / Period	Mean diagonal	Upward from lowest state	Downward from highest state
Relative to national mean (0.75/1.00/1.25), 2011–2023	0.724	0.260	0.247
Absolute thresholds (20/30/40 beds), 2011–2023	0.717	0.284	0.379
Fixed 2011 quartiles (12.8/17.2/23.3 beds), 2011–2023	0.590	0.393	0.056
Relative scheme, 2011–2017 only	0.677	0.255	0.304
Relative scheme, 2018–2023 only	0.751	0.333	0.130

The spatial Markov chain (Appendix Table A3) conditions transitions on the state of a province’s neighbors. Point estimates suggest that upward mobility from the lowest state is rarer when neighbors are also low (0.100) than when neighbors are in the middle states (0.282–0.316), but the homogeneity tests do not reject equality of the conditional matrices (Q = 26.3, *p* = 0.558; LR = 27.4, *p* = 0.497). Neighborhood context therefore does not significantly condition mobility, a result consistent with the dissolution of global autocorrelation after 2014. Proposition P3 is supported.

### 5.4 σ- and β-convergence

Absolute β-convergence holds over the full period, with *β* = −0.056 (*p* < 0.001, R^2^ = 0.639) over *T* = 12 years, an implied speed of 9.31% per year and a half-life of ln(2)/0.0931 = 7.4 years (Table 7, Figure 6). Sub-period estimates are −0.134 (2011–2017, *T* = 6) and −0.137 (2018–2023, *T* = 5): nearly identical coefficients on either side of the statistical revision, indicating that convergence is a property of the data rather than an artifact of the break. A province 10% below the national mean grows roughly 0.6 percentage points faster per year over the full horizon and 1.4 points faster within sub-periods.

**Table 7:** Absolute *β*-convergence, cross-sectional estimates.

Sample period	T (years)	*β*	*p*	R^2^	Speed (%/yr)	Half-life (yrs)	*N*
Full, 2011–2023	12	−0.0561***	<0.001	0.639	9.31	7.4	31
2011–2017	6	−0.1337***	<0.001	0.674	27.02	2.6	31
2018–2023	5	−0.1368***	<0.001	0.506	23.04	3.0	31

***p < 0.01. Speed s = −ln(1 + βT)/T; half-life = ln(2)/s.

**Figure 6:**
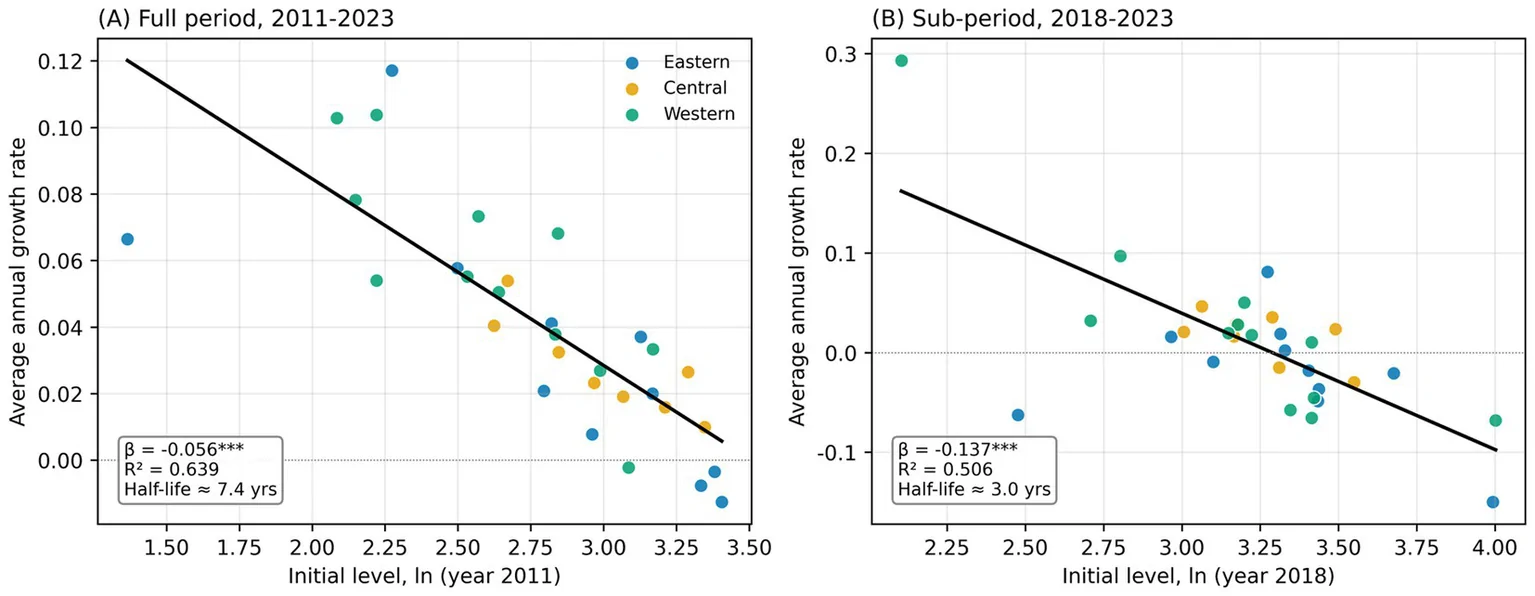
Absolute β-convergence, full and post-revision periods. **(A)** Full period, 2011–2023; **(B)** Sub-period, 2018-2023.

Panel estimates of conditional convergence (Table 8) confirm the pattern: the coefficient on the lagged level is negative and significant in every specification, ranging from −0.208 to −0.426, and the revision dummy enters at −0.121 (*p* < 0.05), providing statistical confirmation of the 2018 break. Table 9 adds the spatial lag of the lagged level under Queen contiguity. The spatial-lag coefficient is small and insignificant in every specification (0.026, −0.004 and 0.051), and the convergence coefficient is unchanged (−0.201, −0.213 and −0.413 with the lag term, against −0.196 and comparable values without it). Convergence is therefore not driven by, and not confounded with, spatial spillovers from neighboring provinces—consistent with the absence of global autocorrelation after 2014. The fixed-effects specifications imply convergence speeds above 50% per year, implausibly fast and a textbook symptom of Nickell bias in a panel with *T* = 13.

**Table 8:** Conditional β-convergence, panel estimates.

Variable	(1)	(2)	(3)	(4)
Lagged level (log)	−0.208*** (0.048)	−0.230*** (0.055)	−0.426*** (0.069)	−0.408*** (0.083)
Per capita fiscal expenditure (log)	—	−0.033 (0.022)	−0.170 (0.252)	−0.025 (0.190)
Resident income (log)	—	0.018 (0.101)	−0.095 (0.633)	0.193 (0.249)
Urbanization rate	—	−0.002** (0.001)	0.004 (0.007)	0.001 (0.008)
Per capita GRP (log)	—	0.104 (0.071)	0.149 (0.144)	0.044 (0.120)
Revision dummy	—	—	—	−0.121** (0.044)
Province fixed effects	No	No	Yes	Yes
Year fixed effects	Yes	Yes	Yes	No
Observations	341	341	341	341
R^2^	0.271	0.282	0.381	0.315

Province-clustered standard errors in parentheses. ***p < 0.01, **p < 0.05, *p < 0.10. Observations are 341 because urbanization is unavailable for 2020.

**Table 9:** Conditional β-convergence with a spatial-lag term (Queen contiguity).

Variable	(1)	(2)	(3)	(4)
Lagged level (log)	−0.196*** (0.049)	−0.201*** (0.054)	−0.213*** (0.057)	−0.413*** (0.065)
Spatial lag of lagged level, W·ln y(t − 1)	—	0.026 (0.058)	−0.004 (0.063)	0.051 (0.137)
Per capita fiscal expenditure (log)	—	—	−0.029 (0.019)	−0.130 (0.225)
Resident income (log)	—	—	0.019 (0.108)	−0.057 (0.578)
Per capita GRP (log)	—	—	0.037 (0.064)	0.129 (0.127)
Province fixed effects	No	No	No	Yes
Year fixed effects	Yes	Yes	Yes	Yes
Observations	372	372	372	372
R^2^	0.265	0.266	0.272	0.375

Province-clustered standard errors in parentheses. ***p < 0.01. Urbanization is omitted so that all 31 provinces × 12 transitions are used.

### 5.5 Club convergence and the eastern divergence

The Phillips–Sul log t regression relaxes the common-steady-state assumption of β-convergence (42). For the full sample the estimated slope is +0.132 with a HAC t-statistic of 0.958, above the −1.65 critical value, so the null of convergence is not rejected: China’s provinces form a single convergence club. Within macro-regions, the central (b̂ = +0.113, *t* = 0.456) and western (b̂ = +0.445, *t* = 1.810) regions converge internally; the eastern region does not (b̂ = −0.533, standard error 0.120, *t* = −4.445). Full regression output for all four samples, including intercepts, standard errors and fit, is reported in Appendix Table A1 so that the sign and magnitude of every coefficient can be verified.

The eastern divergence has an identifiable provincial anatomy. Measured from 2018, after the statistical revision, to 2023, bed supply fell by 53% in Zhejiang (from 54.2 to 25.6 beds), by 27% in Hainan (11.9 to 8.7), by 22% in Guangdong (31.0 to 24.3) and by 17% in Beijing (31.1 to 25.9); it fell moderately in Jiangsu and Hebei (−10 and −9%); it was essentially flat in Tianjin and Shanghai; it rose modestly in Liaoning and Shandong (+8% and +10%); and it rose by 50% in Fujian (26.4 to 39.6). The region’s failure to converge internally is thus the product of a small number of large declines at the top of the 2018 distribution coinciding with one large rise, rather than of uniform drift. We measure from 2018 deliberately: a 2017 baseline would attribute to these provinces declines that belong to the revision (Shandong, for example, fell 18.6% in 2018 and rose thereafter). The LISA results locate the same provinces: Shanghai and Fujian in 2017 and Zhejiang in 2023 are the low–high outliers whose supply fell below their neighbors’. What the data cannot show is why: whether the declines reflect cost pressure, a policy-driven shift toward home- and community-based models, changes in bed utilization, or provincial statistical practice. Section 6 treats these as competing speculations rather than established mechanisms.

A partial test of the substitution hypothesis is nonetheless possible with provincial data on community-based older adult care from the same yearbook, available on a consistent basis from 2020, when statistical coverage of community facilities was expanded (Appendix Table A4). Two measures are relevant: beds in community day-care and full-care facilities, which are already included in the bed-supply numerator, and community older adult care service volume, measured in person-times, which is not. The strong form of the hypothesis—that provinces losing beds replaced them with community facility beds—is not supported: across the 31 provinces the 2020–2023 change in bed supply is positively, not negatively, correlated with the change in community facility beds (Spearman ρ = 0.51, *p* = 0.003), and in the east community beds fell by 12.5% alongside total supply, with Zhejiang losing 59% of its community beds. The weak form—that eastern care systems are becoming service-based rather than bed-based—is consistent with the data: community service volume rose by 108% in the east between 2020 and 2023 (306% in the center, 7% in the west), and in 2023 the eleven eastern provinces delivered 54% of national community service person-times while holding 46% of community beds. But this service expansion is not concentrated where beds fell most: the cross-province correlation between the change in bed supply and the change in service volume is small and insignificant (ρ = 0.19, *p* = 0.31), Fujian raised both beds and service volume, and Beijing, Shanghai and Guangdong raised service volume by 150–770% while losing 4–20% of bed supply. The evidence therefore rules out bed-for-bed substitution, is compatible with a broader transition toward service-based provision in the east, and does not identify that transition as the cause of the bed decline. The short window, the 2020 coverage change and the sensitivity of person-time counts to reporting practice all counsel caution.

### 5.6 Dynamic panel estimation as an upper-bound check

Table 10 reports the two-step system GMM estimates. The lagged-level coefficient is 0.794 (*p* < 0.001), implying an annual convergence rate of −ln(0.794) ≈ 23.1% and a half-life of 3.0 years. The AR(2) test (*p* = 0.313) shows no second-order serial correlation in the differenced residuals, the AR(1) test is marginally significant (*p* = 0.099), in the expected direction though less sharply than is typical of first-differenced residuals, and the instrument count (19) is below the number of groups (31). The Hansen test does not reject the overidentifying restrictions at the 5% level (*p* = 0.059), but its proximity to that threshold is a genuine concern about instrument validity that we do not wish to understate. We therefore do not treat the GMM speed as the preferred estimate. Three considerations argue for treating it as an upper bound: the marginal Hansen statistic; the fact that bed supply is a slow-moving stock, so that a three-year half-life is difficult to reconcile with construction and planning cycles; and the closeness of the GMM rate to the sub-period cross-sectional rates (23–27%), which are themselves inflated by the short horizons over which they are computed. Read together, the full-sample cross-sectional rate of 9.3%, the sub-period rates of 23–27% and the GMM rate of 23% bracket the true speed; the robust conclusion is that convergence is present and economically substantial, not that it proceeds at any particular rate. Proposition P4 is supported in this qualified form.

**Table 10:** Two-step system GMM estimates.

Variable	Coefficient	Corrected SE	z	*p*
Lagged level (log)	0.794***	0.084	9.455	<0.001
Per capita fiscal expenditure (log)	−0.036	0.037	−0.956	0.339
Resident income (log)	0.006	0.127	0.050	0.960
Urbanization rate	−0.002	0.002	−1.469	0.142
Per capita GRP (log)	0.100	0.088	1.132	0.258
Time dummies	Yes			
Observations / groups	310/31			
Instruments	19			
AR(1) / AR(2) *p*-value	0.099/0.313			
Hansen test *p*-value	0.059			

Collapsed instruments; Windmeijer-corrected standard errors. ***p < 0.01.

### 5.7 Correlates of bed-supply levels

Table 11 reports correlates of bed-supply levels. The aging rate is excluded for the reason given in Section 3.1 and replaced by the child dependency ratio, and each specification is estimated both with urbanization (372 observations) and without it (all 403 observations). With province fixed effects only, per capita fiscal expenditure is positively associated with supply (8.368, not significant; 10.008, *p* < 0.05 on the full sample), and fiscal self-sufficiency enters marginally in one specification. Once year fixed effects absorb common national trends, neither fiscal variable is significant and the expenditure coefficient changes sign. The correct reading is that the association between fiscal spending and bed supply operates almost entirely through the synchronized nationwide expansion of the 2010s rather than through within-province variation net of that trend; these estimates do not constitute evidence for or against fiscal decentralization theory, and no causal interpretation is attached. The child dependency ratio is the one covariate that survives year fixed effects: it is negatively associated with bed supply in all four specifications (−0.59 to −0.69 beds per percentage point, *p* < 0.10), suggesting that provinces with younger age structures—and, plausibly, stronger family-care norms and competing fiscal claims from education—supply fewer beds per older person. We read this as suggestive rather than causal. Results are essentially unchanged whether urbanization is included, confirming that the 2020 missing values do not affect the conclusions.

**Table 11:** Correlates of older adult care bed supply.

Variable	(1) Province FE	(2) Two-way FE	(3) Province FE	(4) Two-way FE
Per capita fiscal expenditure (log)	8.368 (4.988)	−7.827 (8.929)	10.008** (4.816)	−4.795 (8.445)
Resident income (log)	13.381 (9.877)	−2.107 (17.075)	10.746 (8.815)	−3.420 (16.971)
Urbanization rate	−0.014 (0.392)	0.243 (0.340)	—	—
Per capita GRP (log)	−6.946 (7.022)	2.610 (7.314)	−6.617 (6.405)	2.546 (6.864)
Fiscal self-sufficiency	0.238** (0.109)	0.136 (0.150)	0.175 (0.109)	0.126 (0.151)
Child dependency ratio	−0.692* (0.387)	−0.590* (0.343)	−0.657* (0.378)	−0.656* (0.348)
Observations	372	372	403	403
R^2^	0.595	0.670	0.604	0.673

Province-clustered standard errors in parentheses. **p < 0.05, *p < 0.10. Columns (3)–(4) omit urbanization and use all observations.

### 5.8 Robustness and the spatial incidence of the 2018 revision

Five further exercises probe the results. First, excluding the four centrally administered municipalities and Tibet leaves β-convergence intact (full period −0.053, *p* < 0.001; post-revision −0.085, *p* < 0.01). Second, the four-region classification separating the Northeast leaves the Dagum structure unchanged (within-region share 26.7% in 2011, 29.7% in 2017, 30.2% in 2023). Third, as shown above, sub-period β estimates on either side of the revision are nearly identical, kernel densities are unimodal within each sub-period, and Markov mobility is bidirectional within each sub-period. Fourth, the spatial-lag term in Table 9 does not alter the convergence coefficient. Fifth, we examined the spatial incidence of the 2018 revision directly (Appendix Table A2). The 2017–2018 change in bed supply averaged −9.8% in the east, −1.0% in the center and −14.1% in the west, and the regional difference is significant by the Kruskal–Wallis test (*p* = 0.046); the largest declines occurred in Tibet (−52.6%), Hainan (−35.0%), Xinjiang (−30.4%), Yunnan (−21.5%) and Beijing (−21.5%), and the change was itself spatially clustered (Moran’s I = 0.208, *p* = 0.028). The revision therefore removed proportionally more beds from frontier and western provinces, presumably where the excluded military-affiliated facilities were concentrated. Two implications follow. Cross-sectional comparisons that straddle 2017–2018 are affected, which is why every statistic in this paper is also reported within sub-periods; and the 2018 jump in the Dagum between-region and transvariation components (Table 2) is in part an artifact of this uneven incidence. Neither the direction nor the significance of any convergence result depends on pooling across the break.

## 6 Discussion

Read together, the results tell a coherent story in three acts. In the first act (2011–2016), a national construction push nearly doubled relative bed supply and began compressing provincial gaps from a Gini of 0.26 toward 0.17; supply in this phase was spatially clustered, with a coastal high–high core in the Yangtze River Delta and a low–low south. In the second act (2013–2017), eastern provinces surged ahead, peaking at 32 beds per 1,000 older persons, while the spatial clustering that had accompanied the early expansion dissolved. In the third act (2018–2023), the east decelerated—its supply falling 18.6% from peak, with Zhejiang, Hainan, Guangdong and Beijing leading the decline—while central and western provinces consolidated, producing both the reversal of the regional hierarchy and the continued decline of overall inequality to a Gini of 0.125, now dominated by overlap rather than by between-region gaps.

The spatial findings deserve emphasis because they change how the convergence result should be read. Equalization in China’s bed supply was not simply a narrowing of the gap between provinces; it was a de-clustering of that gap. In 2013 a province’s supply could be predicted from its neighbors’ with a Moran’s I of nearly 0.5; by 2023 it could no longer be predicted with any statistical confidence. This is what an equalization policy that targets laggards regardless of location should produce, and it is consistent with the interpretation that redistributive transfers and planning targets, rather than diffusion between neighbors, drove the compression. It also explains why the spatial-lag term has no effect on the convergence estimates and why the spatial Markov chain shows no significant neighborhood conditioning: the spatial structure that spatial econometric models are designed to capture had largely disappeared by the second half of the period.

Why did the eastern leaders fall back? We can rule some things out and speculate about others. The decline is not an artifact of the 2018 revision, whose incidence was in fact heavier in the west, and it is not a uniform regional drift, since Fujian rose sharply while its neighbors fell. Four mechanisms are consistent with the remaining evidence, and provincial data cannot discriminate among them. The first is the Baumol cost effect: care is labor-intensive, and in China’s coastal provinces the relevant cost pressure comes less from wages per se than from land prices, which constrain the construction of new residential facilities, and from the reliance of the care workforce on migrant labor whose supply is sensitive to urban living costs (32). The second is structural substitution: several eastern provinces have been early movers toward home- and community-based models, and beds do not register such provision. The partial test in Section 5.5 narrows this hypothesis rather than confirming it—community facility beds fell together with total beds, ruling out bed-for-bed substitution, while non-bed community service volume doubled in the east without concentrating in the provinces that lost most beds. The third is utilization: if occupancy rates in existing facilities are low, as facility-level studies in Beijing have found (15), provinces may rationally slow bed construction without reducing effective capacity. The fourth is provincial statistical practice in the reporting of community facility beds. Each of these would produce the observed pattern; none is directly tested here, and we present them as hypotheses for future work with facility-level or city-level data rather than as findings. What the present evidence does establish is the descriptive fact: in relative bed supply, and in relative bed supply only, the east has fallen from leader to laggard.

This descriptive fact updates the received view of regional advantage, and the comparison with earlier studies requires care. Work based on 2010s cross-sections concluded that eastern provinces held the highest resource levels (17), and an equity assessment of 2020 data continues to emphasize an east-favoring imbalance (20). Our longer panel shows the eastern lead to have been a transitory phase of 2013–2017. Three differences may account for the discrepancy. Timing is the most important: our data run to 2023, and the reversal is a post-2018 phenomenon. Indicator definition matters: studies that aggregate institutions, staff and beds, or that use absolute rather than population-normalized measures, will register the east’s larger absolute stock. And scope matters: because the eastern shift toward non-bed provision would lower a bed-based indicator without lowering broader measures of care capacity, part of the reversal we document may be specific to beds. The reversal is therefore best understood as genuine for the indicator studied and of uncertain generality beyond it.

The third finding tempers the good news. Distributional convergence has coincided with stagnation and recent decline in the level of provision: mean bed supply never reached the 35–40 bed planning target and has fallen since 2021 because the older population grows faster than beds are added. Provinces are becoming more equal around a standard that is itself slipping. Equalization and adequacy are therefore distinct objectives, and success on the first must not be mistaken for success on the second. We do not attempt an international benchmark: cross-national comparisons of bed counts are confounded by differences in denominator (60+ versus 65+), in what counts as a bed, and in the balance between institutional and home-based care, and would require a separate study.

Three policy implications follow. First, balanced allocation should be sustained: transfers and capacity-building support should target the provinces that the Markov analysis identifies as persistently occupying the lowest state, wherever they are located, since location no longer predicts supply. Second, aggregate provision must expand at least as fast as the older population if planning targets are to retain meaning. Third, and most important given the limits of the bed indicator, monitoring frameworks should incorporate home- and community-based service capacity alongside beds, so that an eastern province substituting toward non-bed provision is not misread as regressing.

Several limitations should be borne in mind. First, the indicator measures beds in registered institutions and community facilities and nothing else; it does not capture home-based services or insurance coverage, and all conclusions are confined to bed supply. Second, the mechanisms behind the eastern decline cannot be identified with provincial panel data; the partial test of substitution in Section 5.5 rules out bed-for-bed replacement but cannot establish causation, and the remaining explanations are speculative. Third, the correlates analysis is descriptive: coefficients are not robust to year fixed effects, the panel affords limited power, and no causal claims are advanced. Fourth, the 2018 revision changed statistical coverage unevenly across provinces; sub-period estimation mitigates but cannot fully eliminate its effects on cross-sectional comparisons that straddle 2017–2018. Fifth, the provincial scale conceals within-province heterogeneity in accessibility that facility-level studies reveal, and covariates capturing terrain, transport infrastructure and inter-provincial migration were not available on a consistent provincial basis; the resident-versus-registered basis of the older population in the official indicator could likewise not be verified. Sixth, with 31 provinces the asymptotic approximations underlying GMM diagnostics are imperfect, and the marginal Hansen statistic is a reason to treat the GMM speed as a bound rather than a point estimate.

## 7 Conclusion

Drawing on a thirteen-year balanced panel of Chinese provinces and an integrated framework of distributional, spatial and convergence methods, this study asked whether older adult care bed supply has become more equal across provinces. The main findings are as follows: (i) provincial disparities in bed supply narrowed substantially, with the Gini coefficient falling by half from its 2012 peak; the decline was driven by the collapse of net between-region differences, and by 2023 the inequality that remained was dominated by overlap among regional distributions rather than by gaps between them; (ii) bed supply was significantly spatially clustered in the early 2010s and that clustering dissolved as equalization proceeded, so that location no longer predicts supply; (iii) the distribution stayed unimodal with bidirectional mobility under relative and absolute classification schemes alike; (iv) the regional hierarchy reversed, with the east falling from leader in 2017 to laggard by 2023 through large post-2018 declines in Zhejiang, Hainan, Guangdong and Beijing; (v) β-convergence is robust across cross-sectional, panel, spatial-lag and dynamic estimators, with a speed bracketed between roughly 9 and 27% per year; and (vi) equalization coincided with a plateau and recent decline in relative provision that leaves bed supply below national planning targets. Sustaining balanced allocation, expanding aggregate supply in step with population aging, and broadening measurement to encompass home- and community-based services constitute the central priorities for the next stage of older adult care system development in China.

## Data Availability

Publicly available datasets were analyzed in this study. This data can be found here: the source datasets are official statistical publications of the Chinese government and are not hosted in a single repository with accession numbers. Provincial-level indicators are publicly accessible through the National Bureau of Statistics of China data portal: https://data.stats.gov.cn. The primary source for the outcome variable is the China Civil Affairs Statistical Yearbook (Ministry of Civil Affairs of China), complemented by the China Statistical Yearbook and the Finance Yearbook of China; the authors accessed these yearbooks through the EPS China Data platform (https://www.epsnet.com.cn, institutional subscription). The constructed provincial panel and analysis code are available from the corresponding author on reasonable request. Figure 2 sources: Base map: Natural Earth (https://www.naturalearthdata.com) (data in the public domain); national boundary in the South China Sea and Tibet drawn following the Standard Map of China, Ministry of Natural Resources (http://bzdt.ch.mnr.gov.cn; review no. GS(2019)1830).

## Appendix

**Table A1:** Phillips–Sul log t regressions: full output.

Sample	*N*	*T*	Regression obs.	Intercept	Slope b̂	SE(b̂)	*t*-statistic	R^2^	Decision (5%)
Full sample	31	13	9	−1.298	0.132	0.138	0.958	0.055	Convergence not rejected
Eastern region	11	13	9	0.042	−0.533	0.120	−4.445	0.645	Convergence rejected
Central region	8	13	9	−1.146	0.113	0.249	0.456	0.013	Convergence not rejected
Western region	12	13	9	−2.122	0.445	0.246	1.810	0.173	Convergence not rejected

Regression of log(H₁/H_t_) − 2 log(log t) on log t over the final 70% of the sample (*t* = 5 to 13, 9 observations), with Newey–West HAC standard errors (lag 4). One-sided critical value −1.65.

**Table A2:** Spatial incidence of the 2018 statistical revision: change in bed supply, 2017–2018.

Region	Mean change (%)	SD	Minimum	Maximum
Eastern	−9.8	11.0	−35.0 (Hainan)	0.4 (Shanghai)
Central	−1.0	6.8	−12.2 (Shanxi)	8.7 (Anhui)
Western	−14.1	15.4	−52.6 (Tibet)	4.8 (Inner Mongolia)
Test of regional difference	ANOVA *p* = 0.078; Kruskal–Wallis *p* = 0.046			
Spatial autocorrelation of the change	Moran’s I = 0.208, *p* = 0.028 (Queen contiguity)			

**Table A3:** Spatial Markov chain: transition summary conditional on neighbors’ initial state.

Neighbors’ state	Mean diagonal	Upward from lowest state	Downward from highest state
Low	0.725	0.100	0.000
Medium-low	0.659	0.282	0.286
Medium-high	0.749	0.316	0.296
High	0.672	0.200	0.167
Homogeneity tests	Q = 26.27 (*p* = 0.558); LR = 27.39 (*p* = 0.497)		

Provinces and their spatial lags classified with the same relative cut-offs (0.75, 1.00, 1.25) under Queen contiguity.

**Table A4:** Community-based older adult care and the change in bed supply, 2020–2023.

Region	Change in community facility beds (%)	Change in community service person-times (%)	Share of national community beds, 2023 (%)	Share of national service person-times, 2023 (%)
Eastern	−12.5	107.6	45.5	54.1
Central	3.0	305.7	26.6	36.2
Western	−10.0	7.0	27.8	9.7
Cross-province correlation with change in bed supply (Spearman ρ)	0.51 (*p* = 0.003)	0.19 (*p* = 0.31)		

Community facility beds are the sum of community day-care and full-care beds; both are included in the bed-supply numerator. Service person-times are not. Source: China Civil Affairs Statistical Yearbook; statistical coverage of community facilities was expanded from 2020, so earlier years are not comparable.
